# Reduction of Secreted Frizzled-Related Protein 5 Drives Vascular Calcification through Wnt3a-Mediated Rho/ROCK/JNK Signaling in Chronic Kidney Disease

**DOI:** 10.3390/ijms21103539

**Published:** 2020-05-17

**Authors:** Yun Jung Oh, Hyunsook Kim, Ae Jin Kim, Han Ro, Jae Hyun Chang, Hyun Hee Lee, Wookyung Chung, Hee-Sook Jun, Ji Yong Jung

**Affiliations:** 1 Department of Internal Medicine, Graduate School of Medicine, Gachon University, Incheon 21936, Korea; lusallome21@hanmail.net; 2 Division of Nephrology, Department of Internal Medicine, Cheju Halla General Hospital, Jeju 63127, Korea; 3 Division of Nephrology, Gachon Advanced Institute for Health Sciences and Technology, Incheon 21999, Korea; toyjoy1@nate.com; 4 Division of Nephrology, Department of Internal Medicine, Gil Medical Center, Incheon 21565, Korea; dofdnc@naver.com (A.J.K.); goddard@hanmail.net (H.R.); jhchang@gilhospital.com (J.H.C.); lhh90@hanmail.net (H.H.L.); jwkpsj79@hanmail.net (W.C.); 5 Division of Nephrology, Department of Internal Medicine, College of Medicine, Gachon University, Incheon 21565, Korea; 6 College of Pharmacy, Gachon University, Incheon 21936, Korea; hsjun@gachon.ac.kr; 7 Lee Gil Ya Cancer and Diabetes Institute, Gachon University, Incheon 21999, Korea

**Keywords:** secreted frizzled-related protein 5, Wingless-related integration site, vascular calcification, chronic kidney disease

## Abstract

Vascular calcification (VC) is commonly associated with bone loss in patients with chronic kidney disease (CKD). The Wingless-related integration site (Wnt) regulates osteoblast activation through canonical signaling pathways, but the common pathophysiology of these pathways during VC and bone loss has not been identified. A rat model of adenine-induced CKD with VC was used in this study. The rats were fed 0.75% adenine (2.5% protein, 0.92% phosphate) with or without intraperitoneal injection of calcitriol (0.08 µg/kg/day) for 4 weeks. Angiotensin II (3 µM)-induced VC was achieved in high phosphate medium (3 mM) through its effect on vascular smooth muscle cells (VSMCs). In an mRNA profiler polymerase chain reaction assay of the Wnt signaling pathway, secreted frizzled-related protein 5 (sFRP5) levels were significantly decreased in the CKD rat model compared with the control group. The repression of sFRP5 on VSMC trans-differentiation was mediated through Rho/Rho-associated coiled coil containing protein kinase (ROCK) and c-Jun N-terminal kinase (JNK) pathways activated by Wnt3a. In a proof of concept study conducted with patients with CKD, serum sFRP5 concentrations were significantly lower in subjects with VC than in those without VC. Our findings suggest that repression of sFRP5 is associated with VC in the CKD environment via activation of the noncanonical Wnt pathway, and thus that sFRP5 might be a novel therapeutic target for VC in CKD.

## 1. Introduction

Chronic kidney disease mineral and bone disorder (CKD-MBD) is a common complication and among the important contributors to cardiovascular mortality in patients with CKD [1,2]. Abnormalities in calcium, phosphorus, parathyroid hormone, and vitamin D metabolism contribute to progression of bone loss and vascular calcification (VC) [3]. With the progression of CKD, decreases in bone mass and VC progress simultaneously [4,5], which is suggestive of a strong relationship between bone loss and VC.

The Wingless-related integration site (Wnt) signaling pathway is considered to be a critical component of the regulation of bone formation and resorption, and study of this pathway has increased our understanding of the pathophysiology of CKD-MBD. In particular, the canonical Wnt/β-catenin signaling pathway has been of great interest in bone research [6,7], as it is thought to play an important role in the regulation of osteogenesis by inducing the differentiation of mesenchymal precursors toward osteoblasts and promoting the differentiation of osteoblast precursors toward mature osteoblasts while inhibiting osteoblast apoptosis [8]. Therapeutic approaches for osteoporosis that target Wnt/β-catenin signaling have been developed; they aim to increase bone formation by upregulating Wnt/β-catenin signaling via the blockage of endogenous Wnt antagonists, such as sclerostin and Dickkopf-related protein 1, using neutralizing antibodies [9,10].

The Wnt signaling pathway has also been shown to be involved in the pathogenesis of VC [11], which may explain the coexistence of bone and vascular disease. Previous studies have shown that Wnt inhibitors are associated with bone health, vascular disease, and clinical outcomes in patients with CKD [12], supporting a potential role of Wnt signaling in cross-talk between bone loss and VC in CKD-MBD. Most early studies of the role of Wnt signaling in vascular disease revealed the contribution of canonical Wnt/β-catenin signaling [13,14,15]. However, recent data supporting the involvement of noncanonical Wnt signaling in vascular disease have emerged. Studies have demonstrated that the noncanonical Wnt signaling pathway is associated with the differentiation of bone-marrow mesenchymal stem cells in VC [16,17] and involved in the pathogenesis of aortic valve calcification [18]. Despite the emerging evidence implicating Wnt signaling in vascular disease, little is known about the roles of the noncanonical Wnt signaling pathway in VC and bone metabolism.

In this study, we attempted to identify changes in the Wnt signaling system in the context of CKD with VC. We explored the gene expression profile of the Wnt signaling pathway in an animal model of CKD to investigate how regulation of this pathway is altered as VC progresses in CKD. We also identified a candidate for the main regulator of Wnt signaling leading to VC.

## 2. Results

### 2.1. Expression Profiling of Wnt Signaling Pathway Molecules in an Animal Model of Adenine-Induced CKD with VC

To induce CKD with VC, rats were fed a specially formulated adenine diet for 4 weeks. A subset of rats also received intraperitoneal (IP) injections of calcitriol (0.08 ug/kg) daily (Supplemental Figure S1A). The kidneys of all adenine-treated rats showed interstitial fibrosis with abundant deposition of adenine crystals and mononuclear cell infiltration. Only rats on the adenine diet that received IP calcitriol injections developed VC (Supplemental Figure S1B). Kidney function was significantly decreased in both adenine-treated groups compared with controls (Supplemental Figure S1C,D).

To explore how Wnt signaling regulation is altered during the progression of VC in CKD, we explored the gene expression profile of Wnt signaling pathway molecules in the animal model of CKD with VC. Among the 84 genes studied, 13 showed significantly different expression between the CKD with VC and control groups (Supplemental Figure S2). Secreted fizzled-related protein 5 (sFRP5) was highly under-expressed and sFRP4 was over-expressed in the CKD with VC group compared with the control group. Of the numerous candidates involved in the Wnt signaling pathway, sFRP5 expression was decreased in the CKD with VC model compared to the control group. The full list of Wnt signaling–related genes, based on the RT^2^-profiler polymerase chain reaction (PCR) array, is provided in Supplemental Figure S3.

### 2.2. Effect of CKD on RUNX2, sFRP4, and sFRP5 Expression in Vascular Smooth Muscle Cells

**Figure 1:**
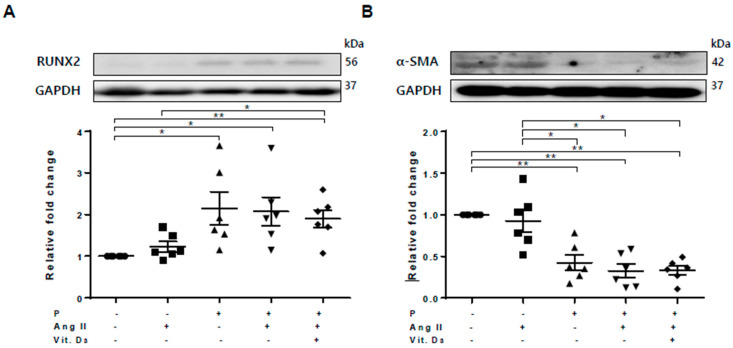
Osteoblastic trans-differentiation of vascular smooth muscle cells (VSMCs) is induced in a chronic kidney disease (CKD) environment. VSMCs were treated with angiotensin II and vitamin D in high-phosphate medium to mimic CKD conditions. Six replicates per condition were performed. The protein levels of Runt-related transcription factor 2 (RUNX2) and α–smooth muscle actin (α-SMA) levels were determined using Western blotting and normalized to glyceraldehyde 3-phosphate dehydrogenase (GAPDH). Bar charts represent the densitometric quantification of immunoblots. (**A**) The expression of RUNX2 was significantly increased in vascular calcification (VC) medium compared with the control; (**B**) The expression of α-SMA was reduced in VC induction medium compared with the control. Data are expressed as means ± standard errors of the mean from six independent experiments. * *p* < 0.05, ** *p* < 0.01.

**Figure 2:**
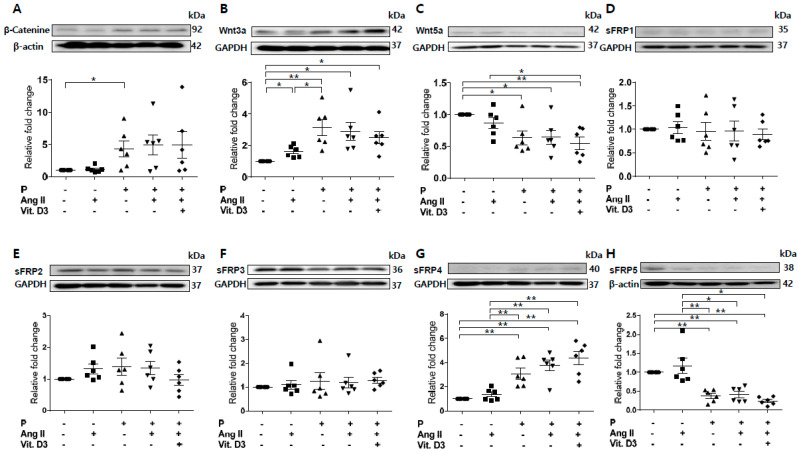
Expression of secreted frizzled-related proteins (sFRPs) and Wnt signaling in vascular smooth muscle cells (VSMCs) exposed to vascular calcification (VC) induction medium (high-phosphate, angiotensin II, and vitamin (D) were measured by Western blotting. Six replicates per condition were performed. The expression levels of β-catenin (**A**) and Wnt3a (**B**) were significantly increased and Wnt5a (**C**) expression was decreased in VC induction medium compared with the control. The expression levels of sFRP1−3 (**D**–**F**) were not affected by the chronic kidney disease environment. The expression of sFRP4 (**G**) was increased and that of sFRP5 (**H**) was decreased in VC induction medium compared with the control. Data are expressed as means ± standard errors of the mean from six independent experiments. * *p* < 0.05, ** *p* < 0.01.

Based on the RNA profile and PCR array, we investigated sFRP5 regulation in CKD with VC in vitro. Vascular smooth muscle cells (VSMCs) were treated with angiotensin II and vitamin D in high phosphate medium (VC induction medium) to mimic the CKD with VC environment. Osteoblastic differentiation of VSMCs was demonstrated by elevated expression of the osteoblast marker Runt-related transcription factor 2 (RUNX2) and decreased expression of the smooth muscle lineage marker α–smooth muscle actin (α-SMA; Figure 1). All sFRPs (sFRP1−5) were expressed in VSMCs (Figure 2D–H). The expression levels of Wnt5a and sFRP5 were significantly downregulated after exposure to VC induction medium (Figure 2C,H). The expression levels of β-catenin, Wnt3a, and sFRP4 were upregulated under pro-calcifying conditions (Figure 2A,B,G). The expression levels of sFRP1-3 were not affected by the CKD environment (Figure 2D–F).

### 2.3. Effect of sFRP5 on RUNX2 in VSMCs in the CKD Environment

**Figure 3:**
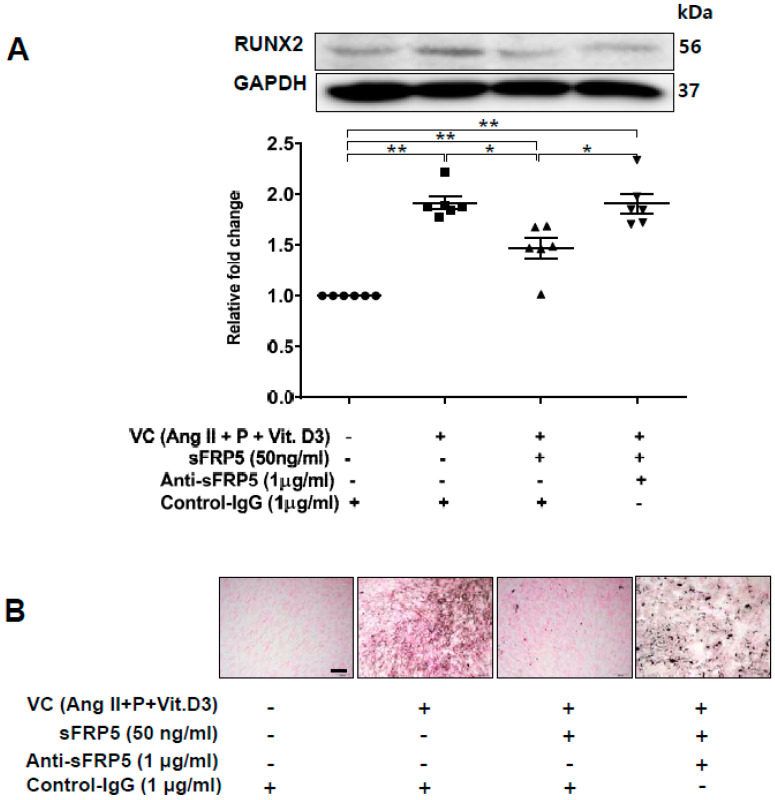
Secreted frizzled-related protein 5 (sFRP5) inhibited osteoblastic trans-differentiation of vascular smooth muscle cells (VSMCs) cultured in vascular calcification (VC) induction media (high-phosphate, angiotensin II, and vitamin (D). The protein level of RUNX2 was determined using Western blotting, and calcification was confirmed visually by von Kossa staining. Six replicates per condition were performed. (**A**) Treatment with sFRP5 of VSMCs in VC induction medium decreased the expression of RUNX2, and neutralization with anti-sFRP5 restored the expression of RUNX2 to control immunoglobulin G levels; (**B**) VSMCs cultured in VC induction medium with different additional interventions and stained with von Kossa stain are shown. Six replicates per condition were performed. VSMCs incubated in VC induction medium showed significantly increased staining compared with the control. Treatment with sFRP5 led to the attenuation of staining, and the addition of anti-sFRP5 resulted in increased staining. Scale bar, 100 µm. Data are expressed as means ± standard errors of the means from six independent experiments. * *p* < 0.05, ** *p* < 0.01.

To explore the functional role of sFRP5 in VC, we induced trans-differentiation of VSMCs via VC induction, added sFRP5, and evaluated the degree of VSMC trans-differentiation. Treatment of VSMCs with sFRP5 in VC induction medium decreased the expression of RUNX2, and neutralization with anti-SFRP5 attenuated the effect of sFRP5 on RUNX2 expression (Figure 3A). In addition, VSMCs were incubated in VC induction medium with different additional interventions and stained using von Kossa staining. Six replicates per condition were performed. VSMCs incubated in VC induction medium showed higher degrees of staining than did the control (Figure 3B). Treatment with sFRP5 resulted in decreased staining, and this inhibitory effect was reversed by anti-sFRP5.

### 2.4. The Protective Effect of sFRP5 against VSMC Differentiation Is Mediated through the Inhibition of Noncanonical Wnt Signaling

**Figure 4:**
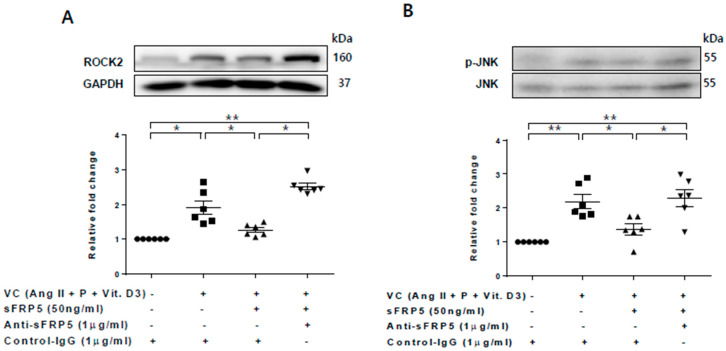
The protective effect of secreted frizzled-related protein 5 (sFRP5) against vascular smooth muscle cell (VSMC) differentiation was mediated by the inhibition of noncanonical (β-catenin–independent) Wnt signaling. VSMCs were cultured with vascular calcification (VC) induction medium in the presence or absence of sFRP5. Six replicates per condition were performed. The protein expressions of ROCK-2 and total and phosphorylated JNK were determined by Western blotting. (**A**) Culture in VC induction medium induced an increase in ROCK-2 expression, and treatment with sFRP5 led to a decrease in ROCK-2 expression. The addition of anti-sFRP5 restored the expression of ROCK-2; (**B**) Culture in VC induction medium increased JNK phosphorylation. Treatment with sFRP5 led to decreased JNK phosphorylation, and neutralization with anti-sFRP5 reversed this effect. Data are expressed as means ± standard errors of the means from six independent experiments. * *p* < 0.05, ** *p* < 0.01.

We next assessed the role of the noncanonical Wnt signaling pathway in the protective effect of sFRP5 against VSMC calcification. Rho-associated coiled coil containing protein kinase-2 (ROCK-2) and phosphorylation of c-Jun N-terminal kinase (JNK), downstream targets of the noncanonical Wnt signaling pathway, were increased in VSMCs incubated in VC induction medium (Figure 4). The addition of SFRP5 significantly decreased the phosphorylation of JNK, and this effect was reversed by neutralization with anti-SFRP5 (Figure 4B). These findings suggest that the protective effect of SFRP5 against the calcification of VSMCs is attributable to the inhibition of the noncanonical Wnt signaling pathway.

### 2.5. Effects of Rho/ROCK and the JNK Pathway on sFRP5 Expression in VSMCs in the CKD Environment

The noncanonical Wnt/planar cell polarity signaling pathway is believed to activate ROCK and JNK through the activation of Rho and Rac, respectively [19,20]. However, previous studies have shown that Rho stimulated JNK activation through ROCK activation [21]. Involvement of the Rho/ROCK/JNK pathway in the trans-differentiation of VSMCs incubated in VC induction medium was confirmed in our study; activation of JNK induced in VC induction medium was inhibited by treatment with Y27632, a ROCK inhibitor (Supplemental Figure S4A). To explore how the Rho/ROCK/JNK signaling cascade affects the expression of sFRP5 in VC, VSMCs were treated with a JNK inhibitor (SP600125) or Y27632 and then exposed to VC induction medium. The protein level of sFRP5 in VSMCs was significantly suppressed by the SP600125 and Y27632 treatments (Supplemental Figure S4B). Based on these results, VSMCs were incubated in VC induction medium and treated with different concentrations of SP600125 or anisomycin, a JNK agonist. Treatment with the JNK inhibitor downregulated sFRP5 protein levels and that with the JNK agonist upregulated sFRP5 protein levels in a dose-dependent manner (Supplemental Figure S5).

### 2.6. Wnt3a-Enhanced Activation of Noncanonical Wnt Signaling Contributes to Osteoblastic Differentiation of VSMCs

**Figure 5:**
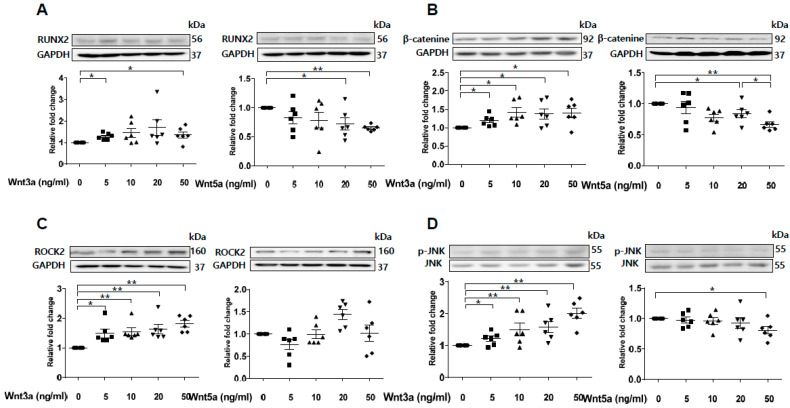
Wnt3a-mediated noncanonical ROCK/JNK signaling is involved in the osteoblastic differentiation of vascular smooth muscle cells (VSMCs). VSMCs were treated with Wnt3a and Wnt5a in a dose-dependent manner. The protein levels of RUNX2, β-catenin, ROCK2, and p-JNK/JNK were determined using Western blotting. Six replicates per condition were performed. (**A**) Wnt3a induced an increase in RUNX2 expression, whereas high-dose Wnt5a (50 ng/mL) induced a decrease in RUNX2 expression in VSMCs; (**B**) Wnt3a upregulated β-catenin protein levels, and Wnt5a dose-dependently downregulated β-catenin protein levels; (**C**) Wnt3a, but not Wnt5a, induced an increase in ROCK2 expression; (**D**) Wnt3a induced JNK phosphorylation, whereas Wnt5a inhibited JNK phosphorylation. Data are expressed as means ± standard errors of the means from six independent experiments. * *p* < 0.05, ** *p* < 0.01.

Previous studies have shown that sFRP5 antagonizes Wnt5a-mediated noncanonical signaling [22,23,24]. However, our experiment showed that Wnt5a was downregulated after exposure to VC induction medium (Figure 2C). Wnt3a was upregulated with the introduction of β-catenin during VC induction (Figure 2B). Thus, we evaluated the effects of Wnt3a and Wnt5a on the osteoblastic differentiation of VSMCs in vitro. Expression of the osteoblastic marker RUNX2 was elevated after stimulation of exogenous Wnt3a in a dose-dependent manner (Figure 5A). In contrast, stimulation with a high dose of Wnt5a (50 ng/mL) led to a decrease in RUNX2 expression. Exogenous Wnt3a induced the upregulation of β-catenin, whereas Wnt5a led to β-catenin downregulation (Figure 5B). Next, we assessed the effects of Wnt3a and Wnt5a stimulation on noncanonical Wnt signaling in VSMCs. Stimulation of Wnt3a led to the upregulation of ROCK-2 and phosphorylation of JNK in a dose-dependent manner, whereas Wnt5a had little effect on the regulation of ROCK-2 and led to the downregulation of pJNK (Figure 5C,D). These results indicate that Wnt3a, rather than Wnt5a, is involved in the activation of noncanonical ROCK/JNK signaling related to the osteoblastic differentiation of VSMCs. This finding suggests that the protective effect of sFRP5 against VSMC differentiation is associated with the inhibition of Wnt3a-mediated noncanonical Wnt signaling.

### 2.7. Expression of RUNX2 and Wnt Signaling–Related Factors in a Rat Model of Adenine-Induced CKD

**Figure 6:**
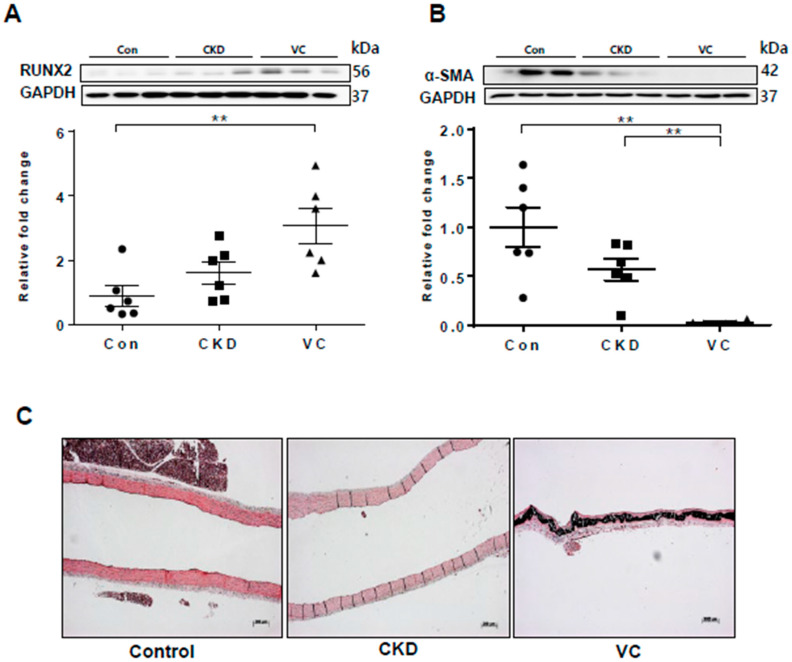
Vascular calcification (VC) was induced in a rat model of adenine-induced chronic kidney disease (CKD). For each model, six rats were included. The protein levels of RUNX2 and α–smooth muscle actin (α-SMA) in rats with adenine-induced CKD were examined using Western blotting; three representative results from each group are presented. VC was confirmed by von Kossa staining of rat aortas. (**A**) The expression of RUNX2 was significantly increased in the CKD with VC group compared with the control group; (**B**) The expression of α-SMA was decreased in the CKD with VC group; (**C**) VC was confirmed by von Koss staining of rat aortas. Scale bar, 200 µm. Data are expressed as mean ± standard errors of the means from six independent experiments. ** *p* < 0.01.

**Figure 7:**
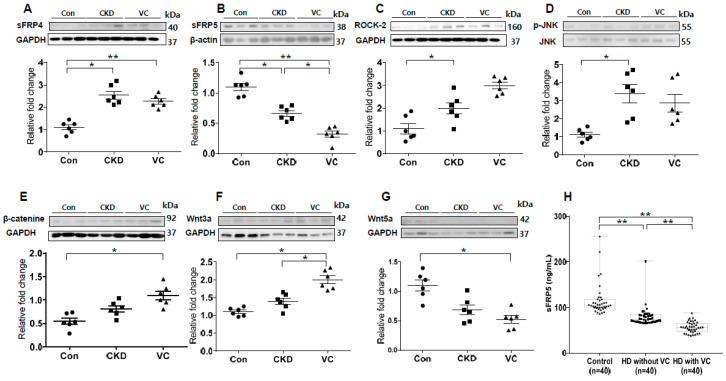
Expression of secreted frizzled-related proteins (sFRPs) and Wnt signaling in an animal model of adenine-induced chronic kidney disease (CKD) were measured by Western blotting, and serum concentrations of sFRP5 were measured in human subjects. For each model, six rats were included; three representative results from each group are presented. The expression of sFRP4 was increased (**A**) and that of sFRP5 was decreased (**B**) in the CKD with vascular calcification (VC) group compared with the control group. The expression levels of ROCK-2 (**C**) and phosphorylation of JNK (**D**) were significantly increased in the CKD group compared with the control group. The expression of β-catenin (**E**) and Wnt3a (**F**) was significantly increased and that of Wnt5a (**G**) was decreased in the CKD with VC group compared with the control group. (**H**) The serum concentration of sFPR5 was significantly lower in patients on hemodialysis (HD) than in subjects with normal renal function. Among patients on HD, subjects with VC had significantly lower levels of serum sFRP5 than did subjects without VC. Three representative samples per group are shown (**A**–**G**). Data are expressed as means ± standard errors of the means from six samples per group. * *p* < 0.05, ** *p* < 0.01.

We next performed an in vivo study using a rat model of adenine-induced CKD to verify the findings of our in vitro studies. The expression of RUNX2, an osteoblastic marker, was significantly increased in the CKD and CKD with VC groups compared with the control groups (Figure 6). VC was confirmed using von Koss staining of the aorta (Figure 6C). The protein level of sFRP4 was increased and that of sFRP5 was decreased in the CKD and CKD with VC groups compared with the control group (Figure 7A,B). The expression levels of ROCK-2 and phosphorylation of JNK, components of the noncanonical Wnt signaling pathway, were significantly increased in the CKD group compared with the control group (Figure 7C,D). β-catenin expression was also increased in the CKD with VC group (Figure 7E). The level of Wnt3a was higher and that of Wnt5a was lower in the CKD with VC group compared with the control group (Figure 7F,G).

### 2.8. Association of the Serum sFRP5 Level with VC in Human Subjects

To investigate whether sFRP5 regulation is relevant for VC in humans, we measured the serum concentrations of sFRP5 in human subjects with normal renal function and in patients on hemodialysis (HD) due to CKD. The serum concentration of sFRP5 was significantly lower in subjects on HD than in those with normal renal function (*p* < 0.01; Figure 7H). Furthermore, among subjects on HD, the serum concentration of sFRP5 was significantly lower in individuals with VC than in those without VC (*p* < 0.01). The baseline characteristics of the subjects, including sFRP5 serum levels, are shown in Supplemental Table S1.

## 3. Discussion

This study showed that the reduction of sFRP5 was associated with the activation of the noncanonical Wnt pathway in an animal model of adenine-induced CKD with VC. In addition, sFRP5 had an inhibitory effect on VSMC trans-differentiation into osteoblast-like cells, which is also involved in the regulation of the Wnt3a-mediated noncanonical signaling pathway through the inhibition of Rho/ROCK/JNK signaling. These findings are suggestive of a suppressive effect of sFRP5 on VC associated with CKD. Moreover, the serum concentration of sFRP5 was significantly lower in patients on HD with VC than in those without VC.

The Wnt signaling pathway is known to be a central regulatory component for bone formation and resorption in the pathogenesis of VC and bone loss [25,26]. Previous studies of VC have focused on the canonical Wnt/β-catenin signaling pathway that promotes it; the activation of Wnt/β-catenin signaling has been detected in calcified human cardiac valves [27,28]. However, with the progression of CKD, Wnt inhibitors such as sclerostin and Dickkopf WNT signaling pathway inhibitor 1 have been shown to be increased [29,30,31,32], leading to the inhibition of canonical Wnt/β-catenin signaling and decreased bone formation. These findings could explain the involvement of Wnt signaling in bone loss in CKD-MBD, but do not explain the mechanism for VC aggravation. Thus, another pathway of Wnt signaling, the noncanonical Wnt signaling pathway, might be regulator of VC in CKD-MBD. Taken together, these findings suggest that two different morphological pathologies of CKD exist simultaneously. Thus, additional research is warranted to identify the switch pathway that determines the involvement of the canonical vs. noncanonical Wnt pathway in CKD.

Several recent studies have documented the involvement of noncanonical Wnt signaling in cardiovascular diseases. Studies have demonstrated the activation of both canonical and noncanonical Wnt signaling in the hypertrophic heart in a mouse model [33], increased expression of noncanonical Wnt ligands in patients with aortic valve calcification, and an association of this increased expression with pathological characteristics [18], suggesting that noncanonical Wnt signaling plays a role in these disease entities. Other investigators have shown that the noncanonical Wnt5a/JNK pathway contributes to cardiac inflammation after ischemia-reperfusion injury, and that sFRP5 diminishes the inflammatory response by antagonizing this pathway [24]. Moreover, a recent study demonstrated that activation of the noncanonical Wnt5a/JNK pathway impaired endothelial vasorelaxation, and that sFRP5 attenuated endovascular dysfunction by antagonizing this pathway [34]. In that study, serum sFRP5 concentration was associated positively with arterial stiffness in patients with type-2 diabetes mellitus, possibly representing a compensatory reaction of sFRP5 to metabolic stress during the early development of atherosclerosis. In our study, sFRP5 and Wnt5a levels were decreased during VC, resulting in increased RUNX expression through activation of the Wnt3a/JNK pathway. sFRP5 levels may increase as a protective mechanism in the early stage of vascular lesion development and decrease in later stages, contributing to lesion development and expansion. The roles of sFRP5, Wnt3a, and Wnt5a in different stages of VC should be studied in future research.

The five members of the sFRP family (sFRP1−5) are known to be endogenous inhibitors of the Wnt signal transduction pathway [35,36]. sFRP5 is a newly identified adipokine that exerts anti-inflammatory effects on metabolic dysfunction in obesity [23]. Numerous studies have supported the protective effect of sFRP5 against various cardiovascular diseases [34,37,38]. Previous in vitro studies have shown that sFRP5 interferes with the canonical Wnt pathway and mitigates high phosphate–induced VC [39]. However, the inhibitory effects of sFRP5 are involved primarily in noncanonical Wnt signaling, and many studies have demonstrated that the effect of sFRP5 is mediated through antagonization of the Wnt5a/JNK noncanonical Wnt pathway [23,24,34,40]. According to the findings of those studies, Wnt5a levels are expected to increase when VC is induced. However, in our experiment, no increase in Wnt5a was observed. On the contrary, Wnt5a levels decreased with the induction of VC, despite the reduction of the sFRP5 level and increased activation of ROCK-2 and JNK signaling. Instead, a significant increase in Wnt3a levels was observed. In our experiment conducted to further evaluate the effects of Wnt3a and Wnt5a on VC, Wnt3a activity led to increased RUNX2 expression and the activation of ROCK-2 and JNK signaling. In contrast, Wnt5 did not induce increased RUNX2 expression or activate ROCK-2 or JNK signaling. These data suggest that Wnt3a activates the noncanonical Wnt signaling pathway to induce VC, and that sFRP5 attenuates VC by inhibiting the Wnt3a/JNK signaling pathway. A previous study showed that sFRP5 suppressed adipogenesis by inhibiting the noncanonical Wnt signaling pathway, in association not with Wnt5a, but instead with Wnt3a [41]. Although Wnt3a is known to be a canonical Wnt ligand, it has been shown to activate noncanonical Wnt signaling [42,43]. These reports support our hypothesis that sFRP5 antagonizes Wnt3a-enhanced noncanonical Wnt signaling, which attenuates VC in the context of CKD.

Our finding of decreased Wnt5a expression during the induction of VC in CKD may be explained by Wnt5a’s regulation of two distinct Wnt signaling pathways. Wnt5a has been shown to inhibit the canonical Wnt signaling pathway and activate the noncanonical Wnt signaling pathway [44,45]. In our experiment, stimulation with Wnt5a led to a considerable decrease in β-catenin expression in VSMCs, supporting the inhibitory role of Wnt5a on canonical Wnt signaling.

The exposure of VSMCs to a uremic milieu resulted in sFRP4 protein overexpression. Our in vivo study yielded similar results, as the induction of CKD and VC increased sFRP4 protein expression, but not mRNA levels. These results are consistent with those of previous studies showing that sFRP4 is involved in renal phosphate wasting with fibroblast growth factor-23 [46]. The signals regulating sFRP4 production, and the physiological role of sFRP4 in VC, need to be further examined.

In this study, we found that serum sFRP5 levels were lower in patients with VC. sFRP5 was found to be highly expressed in retinal pigment epithelial cells and in the pancreas [47]. sFRP5 expression in VSMCs and cardiomyocytes also has been demonstrated [39,48]. However, since Ouchi et al. [23] found that sFRP5 was expressed substantially in adipose tissue, sFRP5 has been considered to be secreted mainly from adipocytes. Initially, sFRP5 was believed to be a secreted protein with autocrine or paracrine function. However, they demonstrated that systemic administration of sFRP5 reversed metabolic dysfunction, suggesting that sFRP5 is detectable in serum and exerts endocrine effects. Thus, we assume that sFRP5 secreted from diverse cells (primarily adipocytes) is released into the systemic circulation and that its serum levels depend on local sFRP5 production. Serum sFRP5 levels were significantly decreased in diabetic and obese patients, and inversely correlated with inflammatory cytokine levels [49,50,51]. Moreover, a previous study demonstrated that hyperglycemia achieved through oral glucose intake decreased circulating sFRP5 levels [49], suggesting that sFRP5 release is influenced by metabolic stress. Based on these findings, we speculate that the reduction of the sFRP5 level in response to CKD progression leads to an increase in noncanonical Wnt signaling and, eventually, VC aggravation. Decreased local production of sFRP5 in the CKD environment may also contribute to the reduction of the serum sFRP5 level. Although the effect of sFRP5 in vascular disease is relatively unexplored, our study suggests that sFRP5 expression changes within calcified vascular tissues, so that not only changes in serum levels of sFRP5 in the CKD environment, but also paracrine effects in or around vascular tissues are affected.

Recent reports claim that even single ischemic events such as myocardial infarction can alter the transcript of blood vessels and play an important role in VSMC remodeling [52,53,54]. Indeed, in the CKD environment, more complex and diverse events can be expected to impact VSMC transcript and remodeling.

Considering the purpose of this study, it is important to discern which Wnt signaling system simultaneously affects VC and bone health. Although we detected an increase in β-catenin expression in our model, we found no other indicator of canonical pathway activation. Thus, in subsequent research we will seek to determine how sFRP5 influences canonical Wnt signaling and conduct a more detailed analysis of the pathway components.

**Figure 8:**
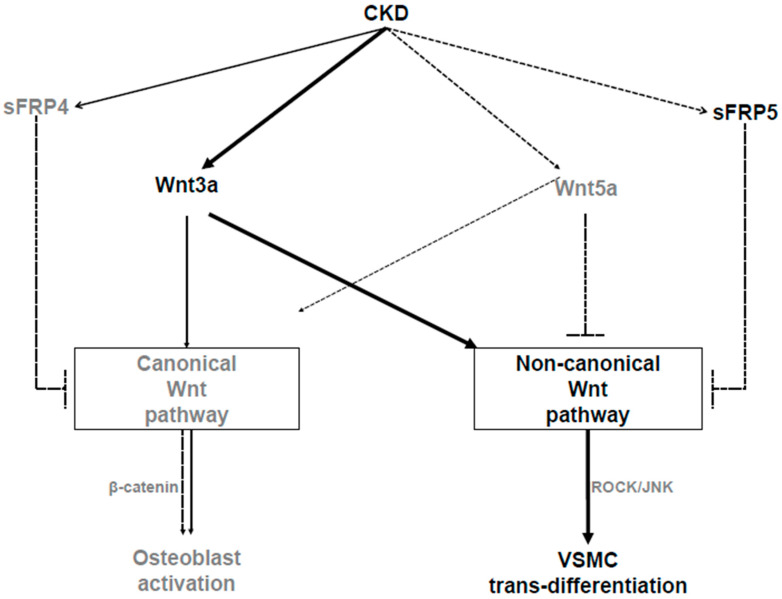
Schematic diagram showing the pathways involved in the pathogenesis of vascular calcification (VC) in chronic kidney disease (CKD). As CKD progresses, secreted frizzled-related protein 5 (sFRP5) and Wnt5a levels decrease, while Wnt3a levels increase. Wnt3a can activate the canonical or noncanonical Wnt signaling pathway. With regard to the pathogenesis of VC, Wnt3a activates noncanonical (ROCK/JNK) Wnt signaling, which leads to the trans-differentiation of vascular smooth muscle cells (VSMCs) into osteoblast-like cells. sFRP5 inhibits Wnt3a-mediated activation of the noncanonical Wnt signaling pathway, leading to the attenuation of the osteoblastic trans-differentiation of VSMCs. Wnt5a inhibits canonical Wnt signaling, which leads to the suppression of osteoblast activation following bone loss in CKD. The solid arrows indicate activation, the dashed lines with blocked ends indicate inhibition, and the dashed arrows indicate reductions in the CKD environment.

In summary, our study showed that the reduction of the sFRP5 level in an animal model of adenine-induced CKD with VC was associated with the activation of the noncanonical Wnt pathway. In addition, sFRP5 had an inhibitory effect on VSMC trans-differentiation into osteoblast-like cells, which is also involved in the regulation of the Wnt3a-mediated noncanonical signaling pathway through the inhibition of Rho/ROCK/JNK signaling (Figure 8). We also demonstrated that serum sFRP5 levels were significantly decreased in patients on HD with VC compared with those without VC. These findings suggest that sFRP5 plays an essential role in the pathophysiology of VC in CKD and might be a therapeutic target for the prevention or treatment of VC.

## 4. Materials and Methods

### 4.1. Cell Culture and Treatment

Human aortic VSMCs were obtained from Lonza Inc. (Walkersville, MD, USA) and grown for six passages in SmGM-2 BulleKit culture medium (Lonza Inc., Walkersville, MD, USA). VSMCs were then transferred to Dulbecco’s modified Eagle’s medium and treated with phosphate to obtain a final phosphate concentration of 3 mM. Angiotensin II (3 mM; Sigma, St. Louis, MO, USA) and calcitriol (1 μg Bonky; Yuyu Pharmaceutical, Seoul, Korea) were added and the cells were incubated for 14 days to induce calcification.

### 4.2. Animal Models

Male Sprague Dawley rats were purchased from Orient Bio (Seongnam City, Gyeonggi Province, Korea). The animals were kept in a light- and temperature-controlled room with free access to chow and deionized water. All animal experiments were performed with the approval of the Institutional Animal Care and Use Committee of Gachon University (LCDI-2018-0136 on 19 December 2018), and in accordance with the Guidelines for Animal Care and Use of the Gachon University Lee Gil Ya Cancer and Diabetes Institute. 

To induce CKD with VC, rats were fed a specially formulated adenine diet for 4 weeks, in a modified version of a previously described adenine model [55]. The adenine diet (Harlan Teklad TD.07239) contained 0.75% adenine with 2.5% protein, 1.06% calcium, and 0.92% phosphorus. In a subset of rats, IP injections of 1,25 (OH)_2_D_3_, calcitriol (0.08 ug/kg Bonky; Yuyu Pharmaceutical) were administrated daily. As the adenine diet alone is unlikely to induce VC [56,57], an appropriate calcitriol concentration was selected to induce VC in all subjects and to avoid overwhelming VC [57,58,59]. Rats in the control group were fed a normal rat diet (PicoLab Rodent Diet 20 5053; Purina, St. Louis, MO, USA) for 4 weeks.

All animals were euthanized 4 weeks after initiation of these diets. Under isoflurane anesthesia, blood samples were collected from the abdominal aorta, and the thoracic aorta was extracted for the analysis of VC and gene expression.

### 4.3. Von Kossa Staining

The rats’ thoracic aortas were fixed in 4% paraformaldehyde and embedded in paraffin blocks. The sections were stained for VC using von Kossa’s method. Briefly, the sections were deparaffinized and washed with distilled water. They were then placed in 5% silver nitrate solution, exposed to sunlight for 20 min, and rinsed with distilled water. The sections were subsequently placed in 5% sodium thiosulfate for 5 min and ten washed with distilled water. Finally, they were counterstained with nuclear fast red for 5 min. Dark brown staining indicated areas of calcification in the vessel walls. Calcification was evaluated semi-quantitatively using Image J software (National Institutes of Health, Bethesda, MD, USA). The percentage of calcified area was calculated as the von Kossa–positive area normalized to the total tissue area, which was measured in 10 representative areas and expressed as a mean value.

### 4.4. Western Blot Analysis

Western blot analysis was performed using anti–ROCK-2 (1:500; Santa Cruz Biotechnology, Inc., Dallas, TX, USA), anti–β-catenin (1:1000; Cell Signaling Technology, Danvers, MA, USA), anti-RUNX2 (1:500; Abcam), anti–p-SAPK/JNK (1:1000; Cell Signaling Technology), anti-SAPK/JNK (1:1000; Cell Signaling Technology), anti-SMA (1:500; Santa Cruz), anti-Wnt5a (1:1000; Abcam), anti-Wnt3a (1000; Millipore), anti-sFRP5 (1:2000; Thermo Fisher), anti-sFRP4 (1:500; Santa Cruz), anti-sFRP3 (1:500; Santa Cruz), anti-sFRP2 (1:500; Santa Cruz), anti-sFRP1 (1:500; Santa Cruz), and anti-GAPDH (1:5000; Flarebio, College Park, MD, USA) antibodies. Blots were immunolabeled using horseradish peroxidase-conjugated secondary antibody and incubated with chemiluminescent substrate (Pierce ECL Plus Western Blotting Substrate; Thermo). The Western blots were quantified using ImageJ software (National Institutes of Health).

### 4.5. RNA Profiler PCR Array

Total RNA from rat aortic tissue was extracted using the PureLink RNA Mini Kit (Ambion). cDNA was synthesized from 0.5 μg RNA using the RT First Strand Kit (Qiagen, Hilden, Germany). For real-time PCR, each reverse-transcription reaction product was mixed with RT^2^ SYBR Green Master Mix (Qiagen) and dispensed into the RT^2^-profiler PCR array (Qiagen). Each 96-well plate contained 84 Wnt signaling–related genes, five housekeeping genes (Actb, B2m, Hprt1, Ldha, and Rplp1), one genomic DNA control, three reverse transcription controls, and three PCR controls.

### 4.6. Human Subjects and Measurement of VC

To measure serum concentrations of sFRP5, blood samples from human subjects with normal renal function (*n* = 40), patients on hemodialysis (HD) without VC (*n* = 40), and patients on HD with VC (*n* = 40) were taken. VC was evaluated on plain x-ray of the lateral lumbar spine using a method described previously by Kauppila [60,61]. The presence of VC was defined as a Kauppila VC score >7, and the absence of VC was defined as a Kauppila VC score of zero.

Serum sFRP5 concentrations were assayed and samples were stored at −70 °C shortly after measurement of VC using a human sFRP5 kit (EK1472; Boster Biological Technology, Pleasanton, CA, USA) according to the manufacturer’s instructions.

This study was approved by the Institutional Review Board of the Gachon University Gil Medical Center (GAIRB2013-118, GBIRB2019-250). All procedures were carried out in accordance with the Declaration of Helsinki, as the study involved human participants. Written informed consent was obtained from all participants. The biospecimens and data used were provided by the Gachon University Gil Medical Center Bio Bank.

### 4.7. Statistical Analysis

The results are presented as means ± standard error of the means. Comparisons of means between the two groups were performed using the independent Student’s *t*-test or Mann–Whitney *U* test, and comparisons among multiple groups were performed using one-way analysis of variance or the Kruskal–Wallis test followed by post hoc multiple comparison tests, as appropriate. Statistical analyses were performed using the GraphPad Prism software (ver. 6.0; GraphPad Inc., La Jolla, CA, USA). *p* values < 0.05 were considered to indicate statistical significance.

## Supplementary Materials

The following are available online at https://www.mdpi.com/1422-0067/21/10/3539/s1, Figure S1: Animal model of adenine-induced chronic kidney disease (CKD), Figure S2: Gene expression profile of Wnt signaling pathway molecules in a rat model of adenine-induced chronic kidney disease (CKD), Figure S3: Full list of Wnt signaling–related genes, based on the RT2-profiler polymerase chain reaction array, Figure S4: The Rho/ROCK/JNK signaling cascade is involved in the trans-differentiation of vascular smooth muscle cells (VSMCs) incubated in a chronic kidney disease environment and affects the expression of secreted frizzled-related protein 5 (sFRP5), Figure S5: Upregulation of JNK increases secreted frizzled-related protein 5 (sFRP5) expression and downregulation of JNK decreases sFRP5 expression, Table S1: Baseline characteristics of human subjects.
