# Supplementary material for: Reduction of Secreted Frizzled-Related Protein 5 Drives Vascular Calcification through Wnt3a-Mediated Rho/ROCK/JNK Signaling in Chronic Kidney Disease
Source: Int J Mol Sci. 2020 May 17;21(10):3539. doi: 10.3390/ijms21103539 (PMC7278993; doi:10.3390/ijms21103539)
Supplement: Supplementary file 1 [file ijms-21-03539-s001.pdf]

SUPPLEMENTAL MATERIAL

Reduction of secreted frizzled-related protein 5 drives vascular calcification through Wnt3a-mediated Rho/ROCK/JNK signaling in chronic kidney disease

Yun Jung Oh[1,2*], Hyunsook Kim[3*], Ae Jin Kim[4,5], Han Ro[4,5], Jae Hyun Chang[4,5], Hyun Hee Lee[4,5], Wookyung Chung[4,5], Hee-Sook Jun[3,6,7], and Ji Yong Jung[3,4,5]

[1]Department of Internal Medicine, Graduate School of Medicine, Gachon University, Incheon, Republic of Korea; [2]Division of Nephrology, Department of Internal Medicine, Cheju Halla General Hospital, Cheju, Republic of Korea; [3]Gachon Medical Research Institute, Gachon University Gil Medical Center, Incheon, Republic of Korea; [4]Division of Nephrology, Department of Internal Medicine, Gil Medical Center, Incheon, Republic of Korea; [5]College of Medicine, Gachon University, Incheon, Republic of Korea; [6]College of Pharmacy, Gachon University, Incheon, Republic of Korea; [7]Lee Gil Ya Cancer and Diabetes Institute, Gachon University, Incheon, Korea

Running Title: sFRP5, Wnt3a, and vascular calcification

*These authors contributed equally to this work.

To whom correspondence should be addressed:
Dr. Ji Yong Jung, Division of Nephrology, Department of Internal Medicine, Gachon University Gil Medical Center, Gachon University College of Medicine, 21, Namdong-daero 774 beon-gil, Namdong-gu, Incheon, 21565, Republic of Korea. Tel: +82 32 458 2621; Fax: +82 32 460 3431; E-mail: jyjung@gachon.ac.kr

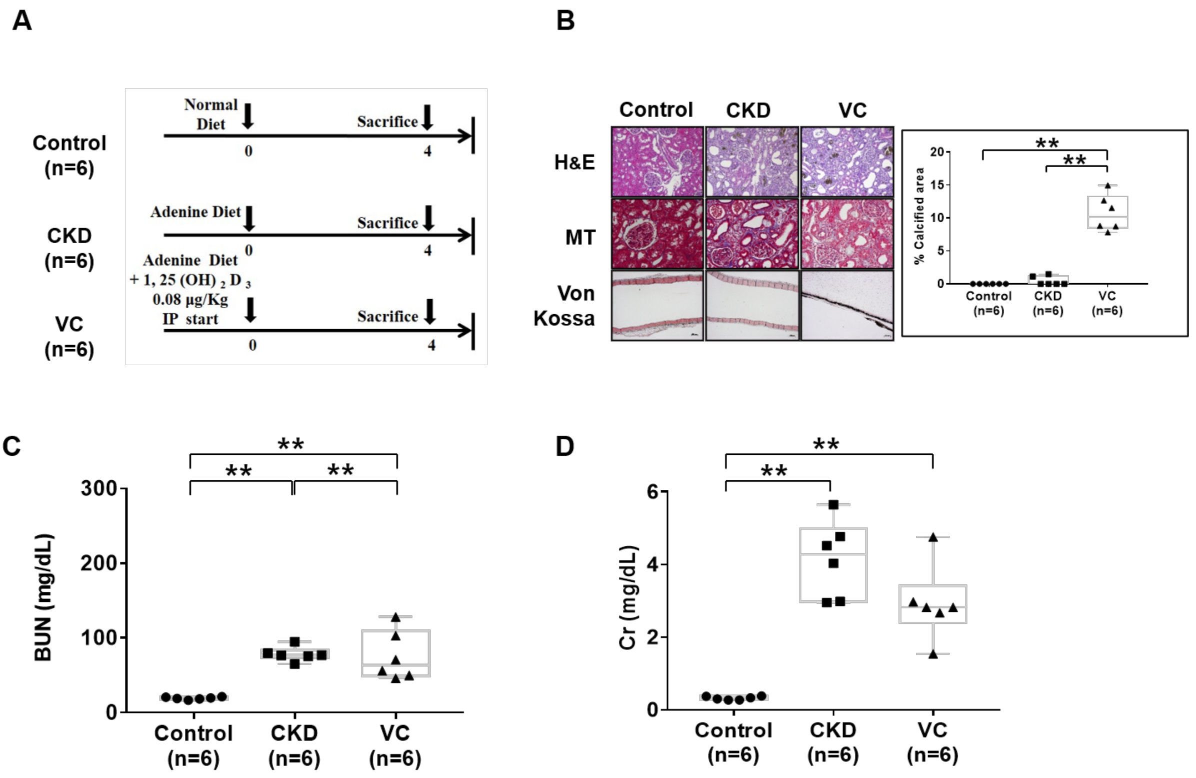

Supplemental Figure S1. Animal model of adenine-induced chronic kidney disease (CKD). (A) Experimental scheme of the rat model of adenine-induced CKD with vascular calcification (VC). Eighteen rats were divided into three groups: the control group (n = 6), the CKD group (n = 6), and the CKD with VC group (n = 6). (B) The kidneys of all adenine-treated rats showed cystic dilatation, degeneration and atrophy of the tubules, interstitial fibrosis, giant cells with abundant deposition of adenine crystals, and mononuclear cell infiltration on hematoxylin and eosin (H&E) and Masson's trichrome (MT) stains. Semiquantitative evaluation of von Kossa–positive areas and representative images of thoracic aorta sections are shown. The aortas of control and CKD rats showed no evidence of VC, whereas rats fed the adenine diet and treated with intraperitoneal calcitriol injections developed VC. (C, D) Blood urea nitrogen and serum creatinine levels were increased significantly in both adenine-treated groups compared with the control group. $^{**}P < 0.01$.

A

ClusterGram

Sample	Dimension	Join Type	Color Coded
Array	2-D	Average	Genes

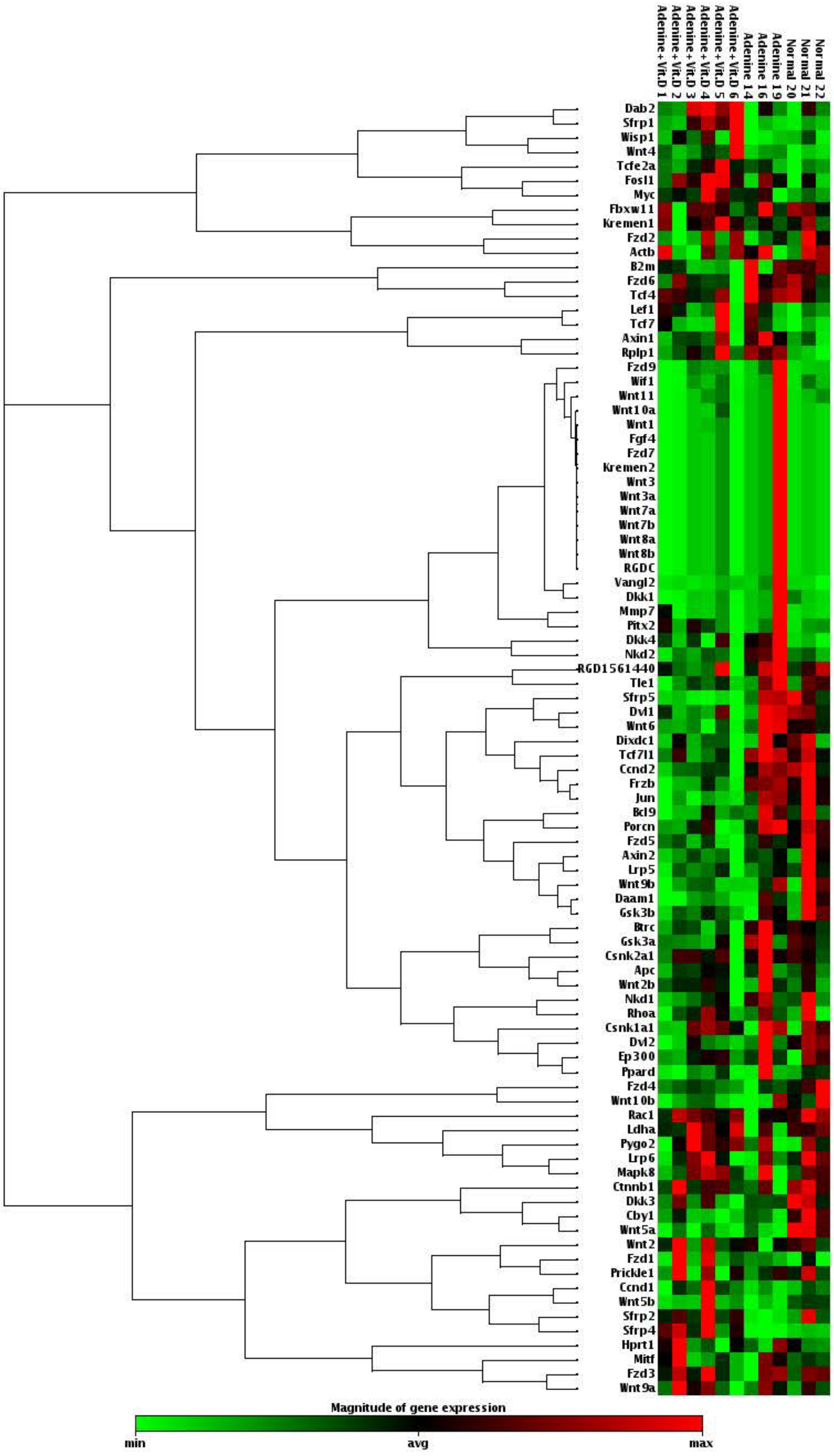

B

Fold regulation comparison and p-value

Control Group	Test Group	Fold Regulation cut off	p-Value cut off
Control Group	Group 1	2	0.05

Position	Gene Symbol	Fold Regulation	p-Value	Comments
A07	Cby1	-2.04	0.026270	A
B06	Dkk4	2.97	0.013447	
D11	Nkd2	2.67	0.015718	
E10	Sfrp4	-5.05	0.002165	A
G04	Wnt5a	-3.29	0.007394	A
G05	Wnt5b	-3.25	0.001962	
H05	Rplp1	2.20	0.000767	

Control Group	Test Group	Fold Regulation cut off	p-Value cut off
Control Group	Group 2	2	0.05

Position	Gene Symbol	Fold Regulation	p-Value	Comments
A07	Cby1	-2.27	0.001972	A
A09	Ccnd2	-2.42	0.010267	
B01	Daam1	-2.47	0.030186	
B12	Fosl1	2.13	0.037202	
C01	Frzb	-3.21	0.005598	
C05	Fzd4	-2.04	0.008685	
C06	Fzd5	-2.37	0.008599	
C12	Jun	-2.80	0.001737	
E10	Sfrp4	4.59	0.023337	
E11	Sfrp5	-6.19	0.001568	A
F09	Wnt10b	-2.56	0.020859	
G04	Wnt5a	-3.60	0.000244	
G06	Wnt6	-2.93	0.003147	

Control Group	Test Group	Fold Regulation cut off	p-Value cut off
Group 1	Group 2	2	0.05

Position	Gene Symbol	Fold Regulation	p-Value	Comments
A09	Ccnd2	-2.27	0.008438	
B06	Dkk4	-2.11	0.036376	
C01	Frzb	-3.52	0.000539	
C12	Jun	-2.73	0.001780	
D11	Nkd2	-3.70	0.001193	
E09	Sfrp2	4.75	0.006719	
E10	Sfrp4	23.20	0.010504	A
E11	Sfrp5	-5.94	0.002867	A
E12	Tcf7l1	-2.47	0.002797	
G06	Wnt6	-3.65	0.010749	

Supplemental Figure S2. Gene expression profile of Wnt signaling pathway molecules in a rat model of adenine-induced chronic kidney disease (CKD). (A) Clustergram based on unsupervised hierarchical clustering shows Wnt signaling–related gene expression patterns in aortic compared with control tissue [control, n = 3; CKD, n = 3; vascular calcification (VC), n

= 6]. The magnitude of gene expression is shown in red (high) and green (low). (B) Hierarchical clustering analysis of the expression of Wnt signaling–related genes by RT^2 Profiler polymerase chain reaction arrays revealed significant differential expression of seven representative genes in the CKD (group 1) and CKD with VC (group 2) groups relative to the control group. Secreted frizzled-related protein 5 (sFRP5) was significantly downregulated in the CKD with VC group compared with the other groups.

Position	RefSeq Number	Symbol	Description
A01	NM_012499	Apc	Adenomatous polyposis coli
A02	NM_024405	Axin1	Axin 1
A03	NM_024355	Axin2	Axin 2
A04	NM_001107703	Bcl9	B-cell CLL/lymphoma 9
A05	NM_001007148	Btrc	Beta-transducin repeat containing
A06	NM_001105969	Vangl2	Vang-like 2 (van gogh, Drosophila)
A07	NM_145676	Cby1	Chibby homolog 1 (Drosophila)
A08	NM_171992	Ccnd1	Cyclin D1
A09	NM_022267	Ccnd2	Cyclin D2
A10	NM_053615	Csnk1a1	Casein kinase 1, alpha 1
A11	NM_053824	Csnk2a1	Casein kinase 2, alpha 1 polypeptide
A12	NM_053357	Ctnnb1	Catenin (cadherin associated protein), beta 1
B01	NM_001108030	Daam1	Dishevelled associated activator of morphogenesis 1
B02	NM_024159	Dab2	Disabled homolog 2 (Drosophila)
B03	NM_001037654	Dixdc1	DIX domain containing 1
B04	NM_001106350	Dkk1	Dickkopf homolog 1 (Xenopus laevis)
B05	NM_138519	Dkk3	Dickkopf homolog 3 (Xenopus laevis)
B06	NM_001109332	Dkk4	Dickkopf homolog 4 (Xenopus laevis)
B07	NM_031820	Dvl1	Dishevelled, dsh homolog 1 (Drosophila)
B08	NM_001172056	Dvl2	Dishevelled 2
B09	XM_001076610	Ep300	E1A binding protein p300
B10	NM_001106993	Fbxw11	F-box and WD repeat domain containing 11
B11	NM_053809	Fgf4	Fibroblast growth factor 4
B12	NM_012953	Fosl1	Fos-like antigen 1
C01	NM_001100527	Frzb	Frizzled-related protein
C02	NM_021266	Fzd1	Frizzled homolog 1 (Drosophila)
C03	NM_172035	Fzd2	Frizzled homolog 2 (Drosophila)
C04	NM_153474	Fzd3	Frizzled homolog 3 (Drosophila)
C05	NM_022623	Fzd4	Frizzled homolog 4 (Drosophila)
C06	NM_173838	Fzd5	Frizzled homolog 5 (Drosophila)
C07	NM_001130536	Fzd6	Frizzled homolog 6 (Drosophila)
C08	XM_006226838	Fzd7	Frizzled family receptor 7
C09	NM_153305	Fzd9	Frizzled homolog 9 (Drosophila)
C10	NM_017344	Gsk3a	Glycogen synthase kinase 3 alpha
C11	NM_032080	Gsk3b	Glycogen synthase kinase 3 beta
C12	NM_021835	Jun	Jun oncogene
D01	NM_053649	Kremen1	Kringle containing transmembrane protein 1
D02	NM_001105767	Kremen2	Kringle containing transmembrane protein 2
D03	NM_130429	Lef1	Lymphoid enhancer binding factor 1
D04	NM_001106321	Lrp5	Low density lipoprotein receptor-related protein 5
D05	NM_001107892	Lrp6	Low density lipoprotein receptor-related protein 6
D06	NM_053829	Mapk8	Mitogen-activated protein kinase 8
D07	NM_001191089	Mitf	Microphthalmia-associated transcription factor
D08	NM_012864	Mmp7	Matrix metallopeptidase 7
D09	NM_012603	Myc	Myelocytomatosis oncogene
D10	NM_001108894	Nkd1	Naked cuticle homolog 1 (Drosophila)

Position	RefSeq Number	Symbol	Description
D11	NM_001107454	Nkd2	Naked cuticle homolog 2 (Drosophila)
D12	NM_019334	Pitx2	Paired-like homeodomain 2
E01	NM_001173355	Porcn	Porcupine homolog (Drosophila)
E02	NM_013141	Ppard	Peroxisome proliferator-activated receptor delta
E03	NM_199396	Prickle1	Prickle homolog 1 (Drosophila)
E04	NM_001106447	Pygo2	Pygopus 2
E05	NM_134366	Rac1	Ras-related C3 botulinum toxin substrate 1
E06	XM_344122	RGD1561440	Similar to nemo like kinase
E07	NM_057132	Rhoa	Ras homolog gene family, member A
E08	NM_001276712	Sfrp1	Secreted frizzled-related protein 1
E09	NM_001100700	Sfrp2	Secreted frizzled-related protein 2
E10	NM_053544	Sfrp4	Secreted frizzled-related protein 4
E11	NM_001107591	Sfrp5	Secreted frizzled-related protein 5
E12	NM_001107865	Tcf7l1	Transcription factor 3
F01	NM_053369	Tcf4	Transcription factor 4
F02	XM_006220666	Tcf7	Transcription factor 7 (T-cell specific, HMG-box)
F03	NM_133524	Tcf3	Transcription factor E2a
F04	NM_001173433	Tle1	Transducin-like enhancer of split 1 (E(sp1) homolog, Drosophila)
F05	NM_053738	Wif1	Wnt inhibitory factor 1
F06	NM_031716	Wisp1	WNT1 inducible signaling pathway protein 1
F07	NM_001105714	Wnt1	Wingless-type MMTV integration site family, member 1
F08	NM_001108227	Wnt10a	Wingless-type MMTV integration site family, member 10A
F09	NM_001108111	Wnt10b	Wingless-type MMTV integration site family, member 10B
F10	NM_080401	Wnt11	Wingless-type MMTV integration site family, member 11
F11	XM_575397	Wnt2	Wingless-type MMTV integration site family member 2
F12	NM_001191848	Wnt2b	Wingless-type MMTV integration site family, member 2B
G01	NM_001105715	Wnt3	Wingless-type MMTV integration site family, member 3
G02	NM_001107005	Wnt3a	Wingless-type MMTV integration site family, member 3A
G03	NM_053402	Wnt4	Wingless-type MMTV integration site family, member 4
G04	NM_022631	Wnt5a	Wingless-type MMTV integration site family, member 5A
G05	NM_001100489	Wnt5b	Wingless-type MMTV integration site family, member 5B
G06	NM_001108226	Wnt6	Wingless-type MMTV integration site family, member 6
G07	NM_001100473	Wnt7a	Wingless-type MMTV integration site family, member 7A
G08	NM_001009695	Wnt7b	Wingless-type MMTV integration site family, member 7B
G09	NM_001106155	Wnt8a	Wingless-type MMTV integration site family, member 8A
G10	NM_001106359	Wnt8b	Wingless-type MMTV integration site family, member 8B
G11	NM_001105783	Wnt9a	Wingless-type MMTV integration site family, member 9A
G12	NM_001107055	Wnt9b	Wingless-type MMTV integration site family, member 9B
H01	NM_031144	Actb	Actin, beta
H02	NM_012512	B2m	Beta-2 microglobulin
H03	NM_012583	Hprt1	Hypoxanthine phosphoribosyltransferase 1
H04	NM_017025	Ldha	Lactate dehydrogenase A
H05	NM_001007604	Rplp1	Ribosomal protein, large, P1
H06	U26919	RGDC	Rat Genomic DNA Contamination
H07	SA_00104	RTC	Reverse Transcription Control
H08	SA_00104	RTC	Reverse Transcription Control
H09	SA_00104	RTC	Reverse Transcription Control
H10	SA_00103	PPC	Positive PCR Control
H11	SA_00103	PPC	Positive PCR Control
H12	SA_00103	PPC	Positive PCR Control

Supplemental Figure S3. Full list of Wnt signaling–related genes, based on the RT^2-profiler polymerase chain reaction array.

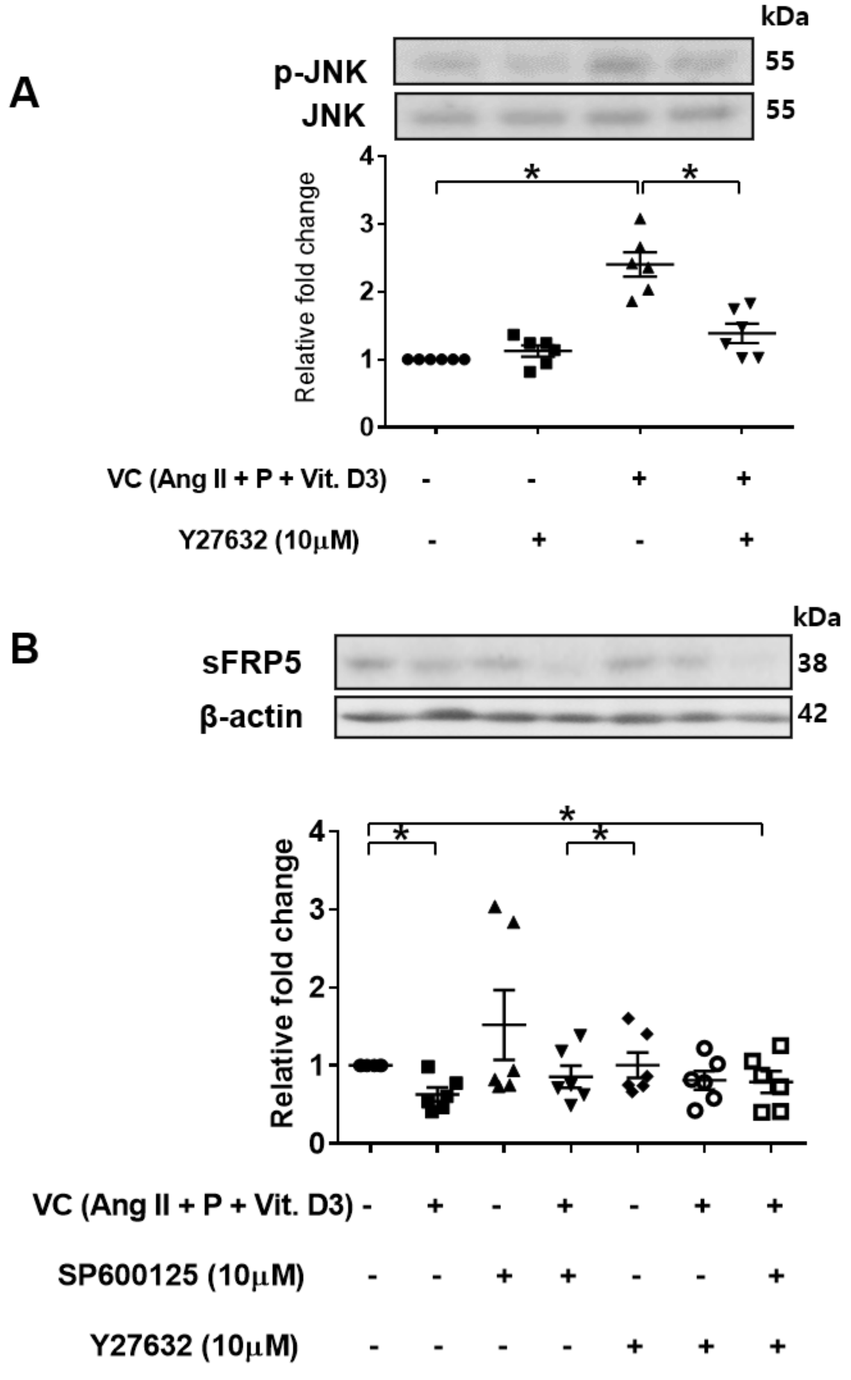

Supplemental Figure S4. The Rho/ROCK/JNK signaling cascade is involved in the trans-differentiation of vascular smooth muscle cells (VSMCs) incubated in a chronic kidney disease environment and affects the expression of secreted frizzled-related protein 5 (sFRP5). (A) VSMCs were cultured with vascular calcification (VC) induction medium in the presence or absence of Y27632 (ROCK inhibitor). The protein levels of total and

phosphorylated JNK were determined by western blotting. Pretreatment with Y27632 attenuated the phosphorylation of JNK induced by the VC induction medium. (B) VSMCs were pretreated with SP600125 (JNK inhibitor) and/or Y27632 and then exposed to VC induction medium. The protein level of sFRP5 was determined by western blotting. Pretreatment with SP600125 and Y27632 resulted in decreased expression of sFRP5. Three replicates per condition were performed. Data are expressed as means ± standard errors of the means from three independent experiments. *$P < 0.05$.

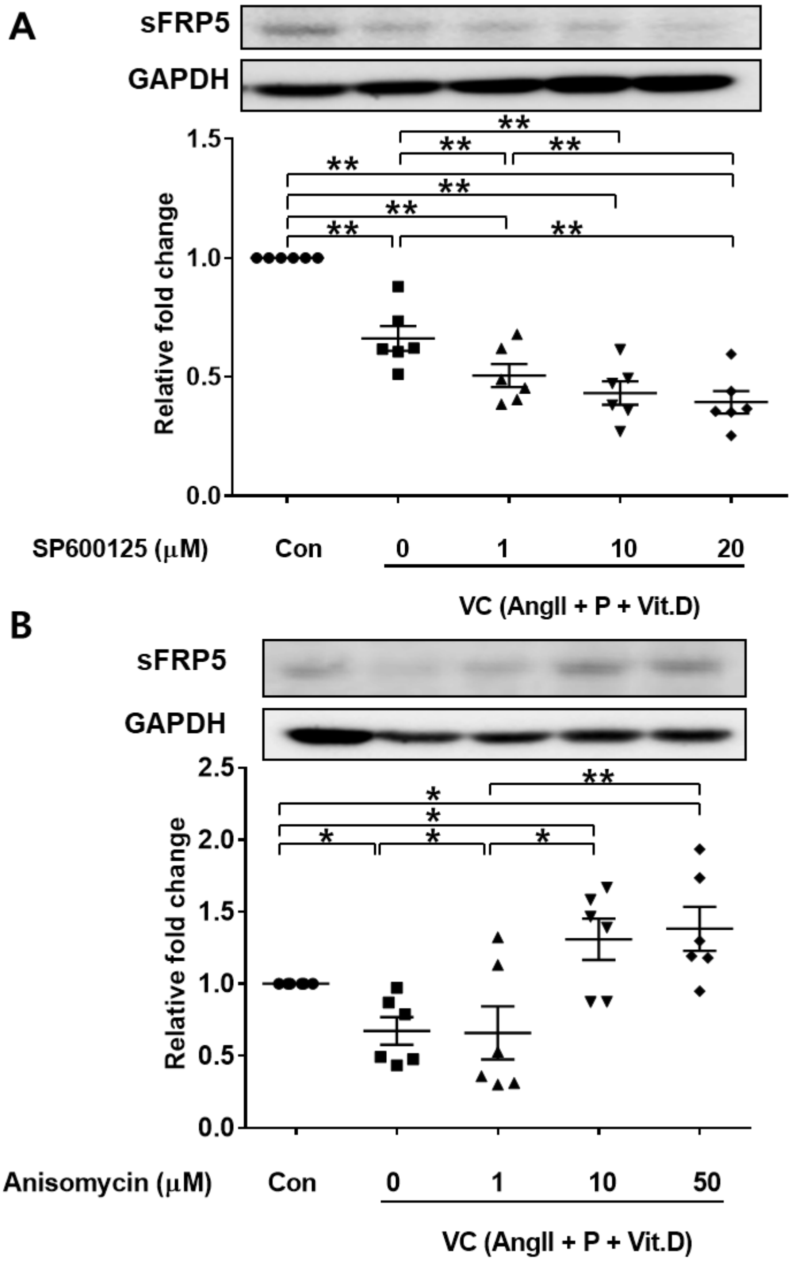

Supplemental Figure S5. Upregulation of JNK increases secreted frizzled-related protein 5 (sFRP5) expression and downregulation of JNK decreases sFRP5 expression. Vascular smooth muscle cells were cultured in vascular calcification induction medium and treated with SP600125 (JNK inhibitor) or anisomycin (JNK agonist) in a dose-dependent manner. Three replicates per condition were performed. The protein levels of sFRP5 were determined by western blotting. (A) SP600125 dose-dependently downregulated sFRP5 protein levels. (B) Anisomycin dose-dependently upregulated sFRP5 protein levels. Data are expressed as means ± standard errors of the means from three independent experiments. *P < 0.05, **P < 0.01.

Supplemental Table S1. Baseline characteristics of human subjects.

	Control (n = 40)	HD patients without VC (n = 40)	HD patients with VC (n = 40)	P*	P†
Age	38.2 ± 11.7	58.0 ± 16.0	58.8 ± 11.6	< 0.001	0.799
Male, n (%)	17 (42.5)	20 (50.0)	21 (52.5)	0.648	1.000
BMI, kg/m^2	23.2 ± 4.0	21.5 ± 3.1	22.4 ± 3.4	0.318	0.199
Smoking	3 (7.5)	1 (2.5)	3 (7.5)	0.536	0.608
Comorbidities					
Hypertension, n (%)	19 (47.5)	30 (75.0)	36 (90.0)	< 0.001	0.141
Diabetes, n (%)	13 (33.3)	16 (40.0)	24 (60.0)	0.045	0.118
CVD, n (%)	1 (2.5)	11 (27.5)	21 (52.5)	< 0.001	0.040
Medications					
ACEi/ARB, n (%)	19 (47.5)	15 (37.5)	26 (65.0)	0.045	0.025
β-blocker, v (%)	1 (2.5)	12 (30.0)	16 (40.0)	< 0.001	0.482
CCB, n (%)	1 (2.5)	18 (45.0)	25 (62.5)	< 0.001	0.178
Diuretics, n (%)	2 (5.0)	26 (65.0)	19 (47.5)	< 0.001	0.176
Statin, n (%)	7 (17.5)	9 (22.5)	12 (30.0)	0.413	0.611
Aspirin, n (%)	1 (2.5)	35 (87.5)	30 (75.0)	< 0.001	0.252
Laboratory					
Hemoglobin, g/dL	13.6 ± 1.7	10.2 ± 1.3	10.6 ± 0.9	< 0.001	0.104
BUN, mg/dL	14.3 ± 4.2	54.3 ± 17.1	54.7 ± 16.7	< 0.001	0.903
Creatinine, mg/dL	0.7 ± 0.1	8.2 ± 2.1	7.7 ± 2.3	< 0.001	0.335
Albumin, g/dL	4.2 ± 0.3	3.8 ± 0.4	3.8 ± 0.4	< 0.001	0.831
Cholesterol, mg/dL	176.0 ± 36.8	134.4 ± 28.9	135.2 ± 27.6	< 0.001	0.900
Calcium, mg/dL	9.1 ± 0.4	8.6 ± 0.7	8.6 ± 0.6	< 0.001	0.893
Phosphate, mg/dL	3.3 ± 0.6	4.3 ± 1.3	4.3 ± 1.3	< 0.001	0.865
hsCRP, mg/dL	0.6 ± 0.9	0.8 ± 2.4	0.5 ± 0.7	0.552	0.457
sFRP5, pg/mL	114.9 ± 34.5	79.1 ± 22.0	56.9 ± 11.9	< 0.001	< 0.001
VC	0.0 ± 0.0	0.4 ± 0.7	11.9 ± 5.2	< 0.001	< 0.001

Abbreviations: CVD, cardiovascular disease; ACEi, angiotensin converting enzyme inhibitor; ARB, angiotensin II receptor blocker; CCB, calcium channel blocker; BUN, blood urea nitrogen; hsCRP, highly selective C-reactive protein; sFRP5, secreted frizzled-related protein 5; VC, vascular calcification score

*Statistical difference (ANOVA) among 3 groups and †comparison test between HD patients without VC and HD patients with VC.
